# Vector time series modelling of turbidity in Dublin Bay

**DOI:** 10.1080/02664763.2024.2315470

**Published:** 2024-02-11

**Authors:** Amin Shoari Nejad, Gerard D. McCarthy, Brian Kelleher, Anthony Grey, Andrew Parnell

**Affiliations:** a Hamilton Institute, Insight Centre for Data Analytics, Maynooth University, Kildare, Ireland; b ICARUS,Department of Geography, Maynooth University, Maynooth, Ireland; c Organic Geochemical Research Laboratory, Dublin City University,DCU Glasnevin Campus, Dublin 9, Ireland

**Keywords:** Bayesian, vector autoregression, turbidity

## Abstract

Turbidity is commonly monitored as an important water quality index. Human activities, such as dredging and dumping operations, can disrupt turbidity levels and should be monitored and analysed for possible effects. In this paper, we model the variations of turbidity in Dublin Bay over space and time to investigate the effects of dumping and dredging while controlling for the effect of wind speed as a common atmospheric effect. We develop a Vector Auto-Regressive Integrated Conditional Heteroskedasticity (VARICH) approach to modelling the dynamical behaviour of turbidity over different locations and at different water depths. We use daily values of turbidity during the years 2017–2018 to fit the model. We show that the results of our fitted model are in line with the observed data and that the uncertainties, measured through Bayesian credible intervals, are well calibrated. Furthermore, we show that the daily effects of dredging and dumping on turbidity are negligible in comparison to that of wind speed.

## 1. Introduction

Studying the variables affecting turbidity is of importance in maintaining coastal ecosystem health. Turbidity is an index for water clarity which measures how suspended solids in water hinder the transmission of light [6]. There are many sources of suspended solids including: phytoplankton; particles from coastal erosion; re-suspended bed sediments; organic detritus from streams; and excessive algae growth [4]. Variability in water turbidity influences the transportation dynamics and distribution of nutrients, contaminants, and biological production [7,11,12,17,24]. Water turbidity is an important habitat factor in many estuarine systems, and changes in it can have a significant impact on management decisions such as the dredging of ports and canals [2].

Our goal in this paper is to evaluate the variations of turbidity in Dublin Bay explained by dredging and dumping operations when controlling for the effect of wind speed, which is an important atmospheric contributor. Dublin has a long history of difficult access for ships to the port area due to sandbanks at the mouth of the port [8]. To solve this problem regular dredging operations have been carried over decades to remove unwanted waste as well as dangerous accumulations of sediments from areas that ships use when entering the port. The excavated materials from the dredging operations are dumped at a more remote location in the bay.

There are relatively few studies focussing on water turbidity in Dublin bay. In one example, [4] used frequentist statistical tests to show that turbidity can be strongly influenced by vessel activity in Dublin bay using data collected from a single location. By contrast, we take a broader approach and look at multiple measuring sites simultaneously corresponding to both the sites where sediment is dumped and dredged, whilst considering issues of turbidity down the water column. We develop a Vector Auto Regressive Integrated Conditional Heteroskedasticity (VARICH) model to control for the spatio-temporal structure using turbidity data measured by five buoys installed at different locations in the bay.

We fit and compare four different models using the turbidity data. The data has many missing values and big gaps for some periods. To fit the models we follow a Bayesian framework to appropriately handle the missingness and infer the parameters of the models using Hamiltonian Monte Carlo (HMC). The purpose of our study is to estimate the effect of covariates on turbidity rather than provide forecasts. The models we fit combine the well-studied approaches Auto Regressive Conditional Heteroskedasticity (ARCH) and Vector Autoregression (VAR). When combined they form a Vector ARCH (or VARCH) model, which we adapt into an integrated model which we name VARICH. A full discussion of these approaches is given below. We show that ARCH-type models perform better for modelling turbidity compared to VAR models that do not account for the heteroscedasticity, and in particular our extended model has the best performance of all. We also show that the daily effects of dredging and dumping on turbidity are negligible in comparison to that of wind speed.

We organise our paper as follows. In Section 2, we describe the data we use in our study. In Section 3, we give a brief introduction to spatiotemporal modelling. In Section 4, we explain our modelling framework. In Section 5, we discuss our findings including plots of the model outputs. We summarise the paper in Section 6 by considering the strengths and weaknesses of our approach and potential areas for future research.

## 2. Data description

**Figure 1.**
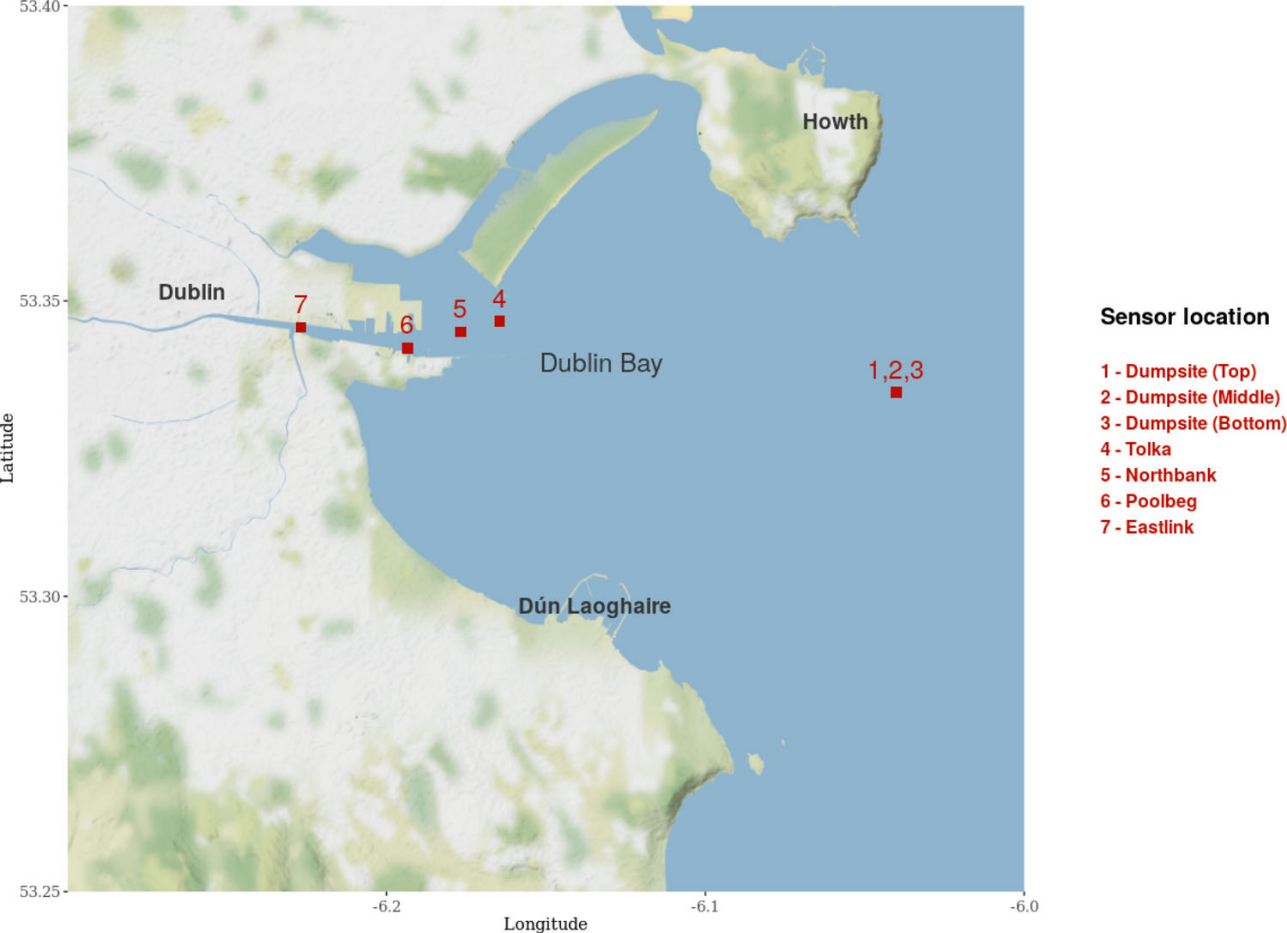
Buoys measuring turbidity in Dublin Bay. We use the same numbering scheme when referring to each site throughout the paper. Buoys 4 to 7 are potential dredging sites, whilst the sediment is dumped at the dumpsite.

Water turbidity levels are measured in Nephelometric Turbidity Units (NTU) which calculate the amount of light reflected through a set of suspended particles. Our dataset contains measurements of water turbidity in NTU at five different locations, four of which take measurements at a single depth and are located throughout the channel from the River Liffey towards Dublin Bay where dredging takes place. The fifth buoy takes measurements at three different levels of the water column and is located approximately 10 km away from Dublin port at the location where the sediments are dumped. Figure 1 shows the locations of the buoys in the bay.

**Figure 2.**
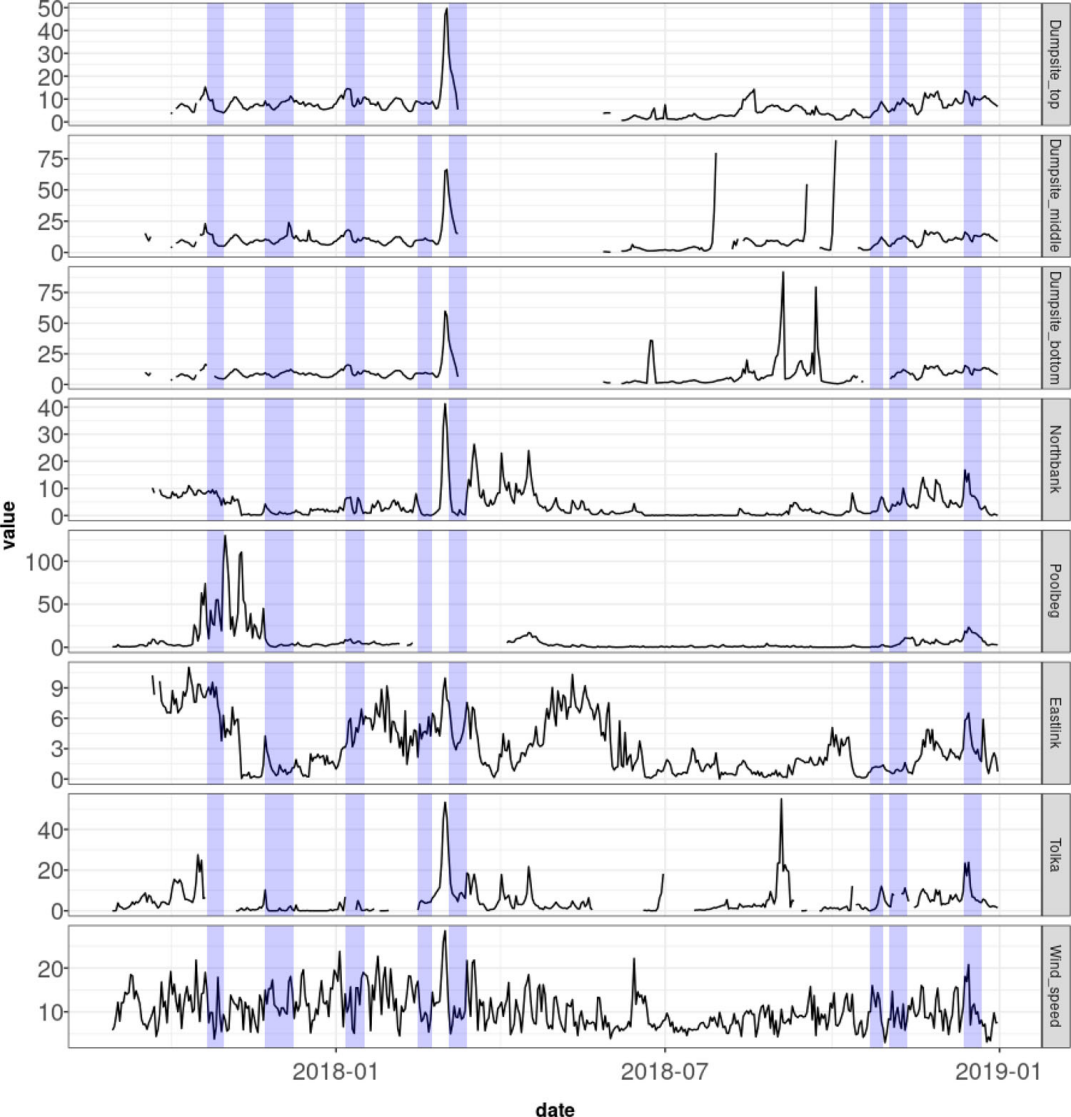
Daily measurements of turbidity (NTU) at Tolka, Eastlink, Poolbeg, Northbank, and various depths of the dumpsite, alongside wind speed (knots) measurements, from 31 August 2017 to 31 December 2018. The highlighted regions indicate the periods during which dredging and dumping operations occurred.

Turbidity measurements are recorded every 15 min by the buoys, but for our analysis we aggregated the raw data into daily averages. This allowed us to focus on the impact of dredging whilst removed short term fluctuations (e.g. that of tides) or the instantaneous impact of vessels arriving or leaving from the port. The aggregation resulted in a total of 488 daily observations per buoy from 31/08/2017 to 31/12/2018. However there are some periods with missing data which seems to be due to equipment failure (e.g. discharged batteries) and gives rise to data gaps when working with our sensor data. A plot of the raw data with missing values is provided in Figure 2. Additionally, we use wind speed data measured at Dublin Airport, provided by [9] for the same period as turbidity data, to control for its effect on turbidity.

## 3. Spatio-temporal models

There are two common approaches to modelling the spatiotemporal structure of data [see e.g. 27, for a review]. One approach involves building a full covariance matrix for each point in space-time and using a multivariate distribution to account for the data generating process. A second approach is to use a multivariate time series model to account for the evolution of a spatial process. The first approach requires matrix operations to be run on a large covariance matrix, and so the second is a useful simplification and commonly used in the applied literature [21]. We similarly found the second approach more suitable for our study in terms of computational efficiency and interpretability due to our data being time rich and space poor. Thus we focus on separable space-time models.

As mentioned above, dynamic spatio-temporal models are a class that are used to model the evolution of a spatial process. Such processes can be continuous in time, but here we focus on cases where time is discrete and the process is given by $\left\{Y_{t}(s):s\in D_{s};t=0,1,\ldots \right\}$. The joint distribution is commonly decomposed using a Markov assumption to give an auto-regressive likelihood of the form $p(Y_{t}(s)|Y_{t-1}(s),\ldots ,Y_{0}(s))=p(Y_{t}(s)|Y_{t-1}(s))$. When the model error is assumed additive (and commonly Gaussian) the model can be written as:

(1) $$\begin{array}{r} Y_{t}=MY_{t-1}+\eta_{t} \end{array}$$

where $Y_{t}$ is a vector of the process values at time *t*, M is the evolution matrix, and $\eta_{t}$ is a vector of spatially white noise processes. Typically the noise processes are assumed to be independent in time [27] and the evolution matrix is assumed to be stationary. Such models are known as Vector Autoregressive (VAR) models, originally introduced by [22] and widely used in macroeconomics, causal inference, and forecasting [1,16,21]. One limitation of the VAR models is their inability to model the heteroscedasticity of the data. To overcome this limitation, it is possible to relax the independence assumption on the noise processes and model their temporal dependence. A very well known approach to model temporal dependence of the noise process is the autoregressive conditional heteroskedasticity (ARCH) model [10] in which the variance of the process is defined as follows:

(2) $$\begin{array}{r} \sigma_{t}^{2}=\alpha_{0}+\sum_{i=1}^{p}\alpha_{i}\epsilon_{t-i}^{2} \end{array}$$

where ${\sigma_{t}}^{2}$ represent the vector of a diagonal covariance matrix applied to $\eta_{t}$, $\alpha_{0}$, and $\alpha_{i}$ are the parameters of the model, and $\epsilon_{t-i}$ are the lagged residuals. The ARCH model is further generalised as the GARCH model [3] which is widely used in finance to model the volatility of financial time series [3]. Furthermore, they have been extended to multivariate time series by considering the covariance matrix of the noise processes and have been used to model non-stationary heteroscedastic data in the spatiotemporal setting [see e.g. 15,19]. In the next section we explain some variations of these models, including the extended model (VARICH) that we use in our study to model turbidity in Dublin bay and infer the effects of dumping, dredging and wind speed on turbidity levels.

## 4. Modelling procedure

In this section we describe the general modelling framework that we follow to build a dynamic spatio-temporal model that describes the response of turbidity to a variety of environmental factors. We then provide specific variations on this template to create four different models which we use for fitting on the data. We denote $Y_{t}$ as an *S*-vector of turbidity measurements at time *t* where *S* is the number of locations (or equivalently buoys), s=1,2,…,S represent the locations and times t (t=1,2,…,T). We write the model hierarchically in two main layers as:

(3) $$\begin{array}{rl} Y_{t}|M_{t},\Sigma_{t} & ∼\mathrm{MVN}\left(M_{t},\Sigma_{t}\right) \end{array}$$

(4) $$\begin{array}{rl} M_{t} & =A+\sum_{j=1}^{P}X_{\mathrm{jt}}\circ \beta_{j}+U_{t} \end{array}$$

where $M_{t}$ is the process mean and $\Sigma_{t}$ is the variance-covariance matrix at time *t*. *A* is an intercept vector, $X_{\mathrm{jt}}$ is an *S*-vector of covariate values associated with covariate j=1,…,P, $\beta_{j}$ is an *S*-vector of fixed effects associated with covariate *j*, and $U_{t}$ is a spatio-temporal structured effect. We use ° to denote the Hadamard product.

The four different structures we consider for fitting the model involve specifying structures for the latent effects $U_{t}$ and the covariance matrix $\Sigma_{t}$. We specify prior distributions associated with these models in the section below following their definition.

Model 1 An ARCH structure with varying $\Sigma_{t}=\mathrm{diag}\{\sigma_{t,1}^{2},\ldots ,\sigma_{t,s}^{2}\}$ and:

(5) $$\begin{array}{rl} \sigma_{t,s}^{2} & =\theta_{1,s}+\theta_{2,s}\epsilon_{t-1,s}^{2} \end{array}$$

(6) $$\begin{array}{rl} U_{t} & =\Phi \epsilon_{t-1} \end{array}$$

with $\Phi =\text{diag}\{\phi_{1},\ldots ,\phi_{s}\}$ being an S×S diagonal matrix of autocorrelation parameters. Here, $\epsilon_{t}=Y_{t}-A-\sum_{j=1}^{P}X_{j,t}\circ \beta_{j}$ and $\epsilon_{t}=[\epsilon_{t,1},\ldots ,\epsilon_{t,s}]$. Model 2 A VAR model with a fixed time-invariant covariance matrix given an inverse-Wishart IW prior:

(7) $$\begin{array}{rl} \Sigma & ∼{\mathrm{IW}}^{-1}(\Psi ,\nu ) \end{array}$$

(8) $$\begin{array}{rl} U_{t} & =\Phi \epsilon_{t-1} \end{array}$$

with *ν* and Ψ as fixed hyper-parameters (we use ν=14 and Ψ=I in our example), and where now Φ is a full rank matrix:

$$\begin{array}{r} \Phi =\left[\begin{array}{ccc} \phi_{1,1} & \ldots & \phi_{1,s}\\ \vdots & \ddots & \vdots \\ \phi_{s,1} & \ldots & \phi_{s,s} \end{array}\right]. \end{array}$$

Model 3 A VARCH model which has both the full rank Φ from model 2 and the time-varying error covariance matrix of model 1. Model 4 A VARICH integrated model that uses the difference in the latent spatio-temporal effects as well as the time-varying error covariance matrix:

(9) $$\begin{array}{r} U_{t}=\Phi (\epsilon_{t-1}-\epsilon_{t-2}) \end{array}$$

where as above the matrix Φ is of full rank.

The posterior distribution for model 4 can be written out in full as follows:

(10) $$\begin{array}{r} p\left(A,\beta ,\theta ,\Phi |Y_{1:T}\right)\propto \left(\prod_{t=3}^{T}p\left(Y_{t}|Y_{t-1};A,\beta ,\theta ,\Phi \right)\right)\times p(A)p(\beta )p(\theta )p(\Phi ) \end{array}$$

To complete the model we need to specify prior distributions for all parameters. We aim to use informative priors for those where we have some degree of information, and use weakly informative and non-informative priors for the remainder. In the below we outline our prior specification for the most complex of the models we fit, model 4, though identical priors were used in the simpler models which corresponds to setting some of the parameter values to zero in a nested model structure.

Our covariates contained in $X_{j,t}$ consist of values associated with dumping and dredging (binary yes/no knowing that the operations happened in the same day, dreding at the dredging sites and dumping at the dumpsite), and wind speed (knots). It is helpful, for prior specification, to consider the regression parameters *β* in terms of their individual scalar components $\left[\beta_{\mathrm{dredge}/\mathrm{dump},s},\beta_{\mathrm{wind},s}\right]$ at site *s*. The full set of priors we used for these values is:

$$\begin{array}{rl} & \beta_{\mathrm{dredge}/\mathrm{dump},s}∼N\left(0,100^{2}\right)\\ & \beta_{\mathrm{wind},s}∼N\left(0,10^{2}\right) \end{array}$$

For the Φ matrix we focus most of the prior mass in the range (-1,1) so that the model selects for stationary behaviour, though non-stationarity can be found if the data are indicative of such phenomena. We thus use:

$$\begin{array}{r} \phi_{\mathrm{ij}}∼N\left(0,0.5^{2}\right) \end{array}$$

For the remaining parameters we set:

$$\begin{array}{rl} A_{s} & ∼N(0,100^{2})\\ y_{1,s} & ∼N(0,100^{2})\\ \theta_{1,s} & ∼TN_{0}(0,1)\\ \theta_{2,s} & ∼\mathrm{Beta}(1,5) \end{array}$$

where $TN_{a}$ refers to the truncated normal distribution with minimum value *a*. All these are expected to be weakly informative, guiding the model towards sensible values whilst letting the data provide the majority of the information. Turbidity in our dataset ranges between 0 and 130 (NTU), so the prior values chosen for *A*, $\beta_{\mathrm{dredge}}$, $\beta_{\mathrm{dump}}$, are considered to be uninformative with respect to this range. The same is true for $\beta_{\mathrm{wind}}$ knowing that wind speed can reach as high as 70 knots during storms, and so a high value of 2 in units of NTU per knot (the units of $\beta_{\mathrm{wind}}$) seems reasonable.

As a final remark on priors we note that many of the turbidity values across sites are missing. We assume that these values are missing at random [MAR; 18] and impute them as part of the model fitting step by treating them as parameters to be estimated. When using the likelihood given above we found that we struggled to produce a posterior with finite variance so we added the extra prior constraint $\;y_{\mathrm{missing}}∼TN_{0}^{100}(0,50^{2})$, a truncated normal between 0 and 100, which seemed to stabilise the missing value estimates.

In summary, model 1 provides a baseline univariate autoregressive model with time changing variance. A more basic constant variance model was also attempted but not shown here due to poor performance. Model 2 tests whether a richer full rank vector mean structure improves the fit at the expense of the changing variance. Model 3 combines both the full vector autoregression with the time changing variance. Finally, model 4 introduces a difference in the latent parameters to capture any potential non-stationarity in the mean. Below we fit each of these models to the data described in Section 2, and use a combination of posterior predictive distributions, information criteria, and plots of the posterior distributions of the parameters to determine the optimal models which we use for interpreting our findings.

## 5. Results

In this section, we report the results of fitting the models described in Section 4 to the turbidity data described in the previous section. We summarise the estimated effects of dredging and dumping operations (recall these are binary variables) and account for the wind speed effect by including the daily wind speed measured in knots. We compare the different models according to their fit to the data, and interpret the best fitting model with a view to obtaining a better understanding of turbidity behaviour in Dublin bay.

### 5.1. Model fitting and comparison

We fit the models using R [20] and the Stan modelling framework [23]. This approach uses Hamiltonian Monte Carlo to update all parameters simultaneously and aims to rapidly converge to the posterior distribution. Through repeated fitting of the models we found that using 1000 iterations, with a warm-up period of 200 iterations, produced acceptable results. We used a MacBook Air equipped with an M1 chip, an 8-core CPU, 8GB of RAM, and 256GB of SSD storage; the computation time was 12 min. We checked convergence using the R-hat diagnostic [5,13] which were all around the target value of 1 at convergence. Details of the estimated parameters are provided in the Appendix 1.

To compare between the models, we use a combination of visual checks where we plot posterior predictions from the model (defined via $M_{t}$) against the true data values, the posterior predictive distribution from the model, and more formal methods. In particular we use the Widely Applicable Information Criterion (WAIC, [26]) and the Leave-One-Out Information Criterion (LOO-IC, [25]) which penalise the likelihood of the model fit based on the complexity of the model. These two information criteria have the added advantage of being easily implemented in R and providing an uncertainty estimate on the value itself. In addition, we also compared VARICH to a frequentist VAR model, details of which are provided in Appendix B.

**Figure 3.**
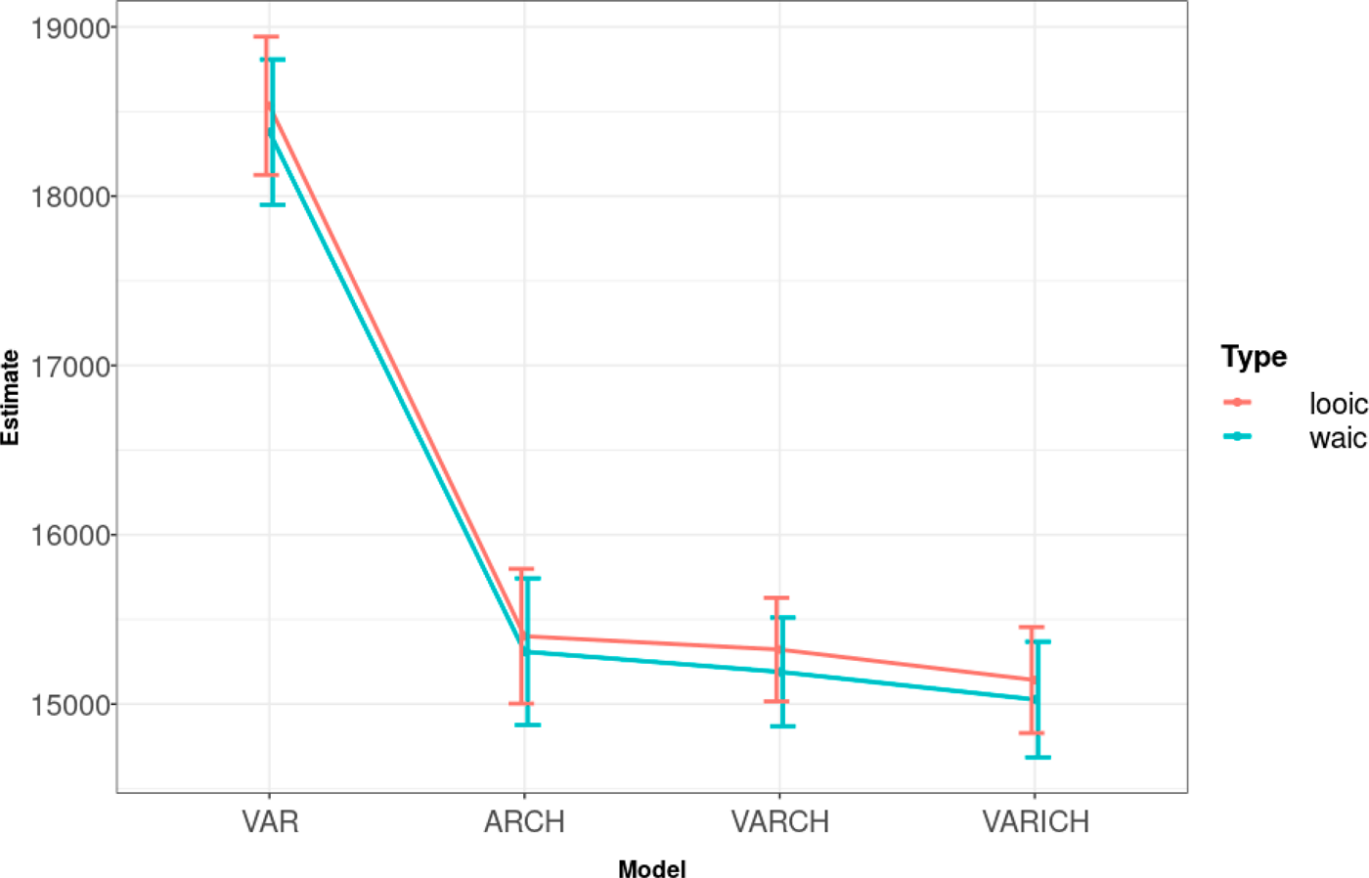
WAIC and LOOIC values for the four fitted models with their associated standard errors.

Figure 3 shows the estimated WAIC and LOOIC values for the four models. The VARICH and VARCH models have the lowest WAIC and LOOIC values indicating better fits. However whilst the mean values of WAIC for the VARICH model are slightly lower there is no clear difference between them. The VARICH model has no extra complexity compared to VARCH, i.e. there are no extra parameters to estimate. Furthermore we computed the spectral radius of the posterior mean of Φ for both models; VARICH gave 0.33 compared to 0.98 for VARCH, which indicates that the VARICH model seems to have removed some of the non-stationarity present in the VARCH formulation. We thus use the VARICH model to create our further results.

**Figure 4.**
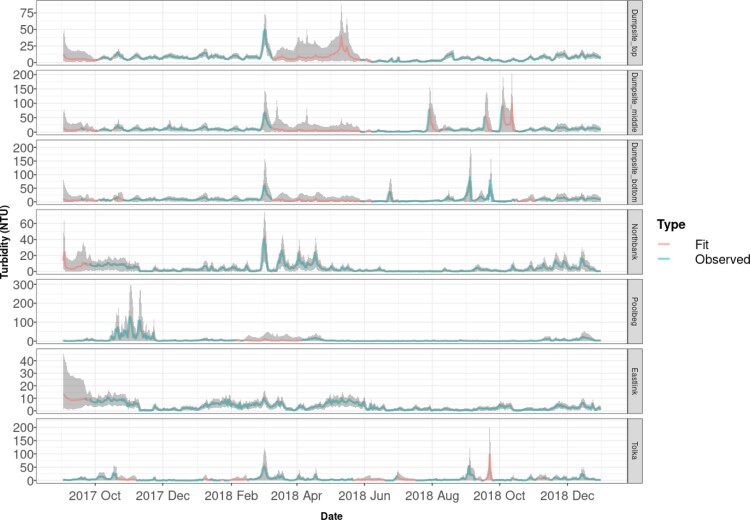
Posterior prediction from the VARICH model vs observed values of turbidity over time for the 7 buoys as labelled. Note the differing vertical axis heights. The shaded periods indicate 95% credible intervals.

Figure 4 shows the posterior prediction of turbidity from the VARICH model against the true data values. The expected values of the fit and the observed values are shown with solid lines coloured in red and blue respectively and the 95% credible intervals are shown with grey bands. As mentioned in Section 2, the dataset has missing periods which are imputed for each location by the model during the fitting process. The vector autoregressive part of the VARICH model allows for drawing information for each site using the available information from the other sites which specifically helps regulate the uncertainty for the missing periods. As expected, the uncertainty during high volatility periods grows as expected through the dynamic structure applied to the variance.

**Figure 5.**
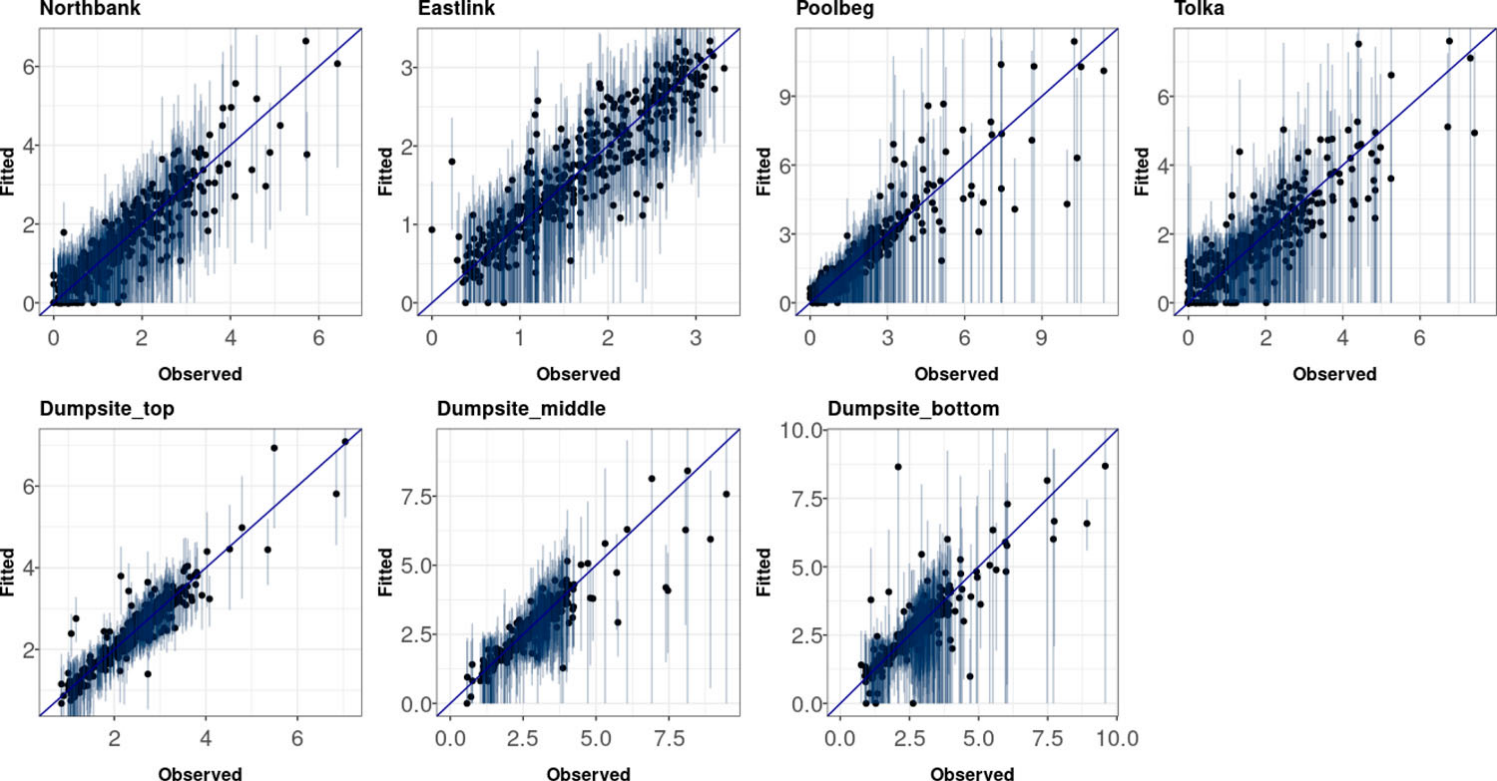
Fitted values from the VARICH model versus observed values of turbidity at different sites. The vertical bars indicate the 95% uncertainty intervals which provide evidence of the coverage properties of the model.

Figure 5 shows the posterior predictive distributions from the VARICH model against the true values with vertical lines indicating the 95% uncertainty intervals. On average the posterior prediction intervals cover 94.6% of the data. The figure shows that the model can successfully retrieve the true values of the turbidity in the dataset with well-calibrated uncertainty estimation at the dumpsite and the dredging sites respectively.

### 5.2. Effects of covariates on turbidity

**Figure 6.**
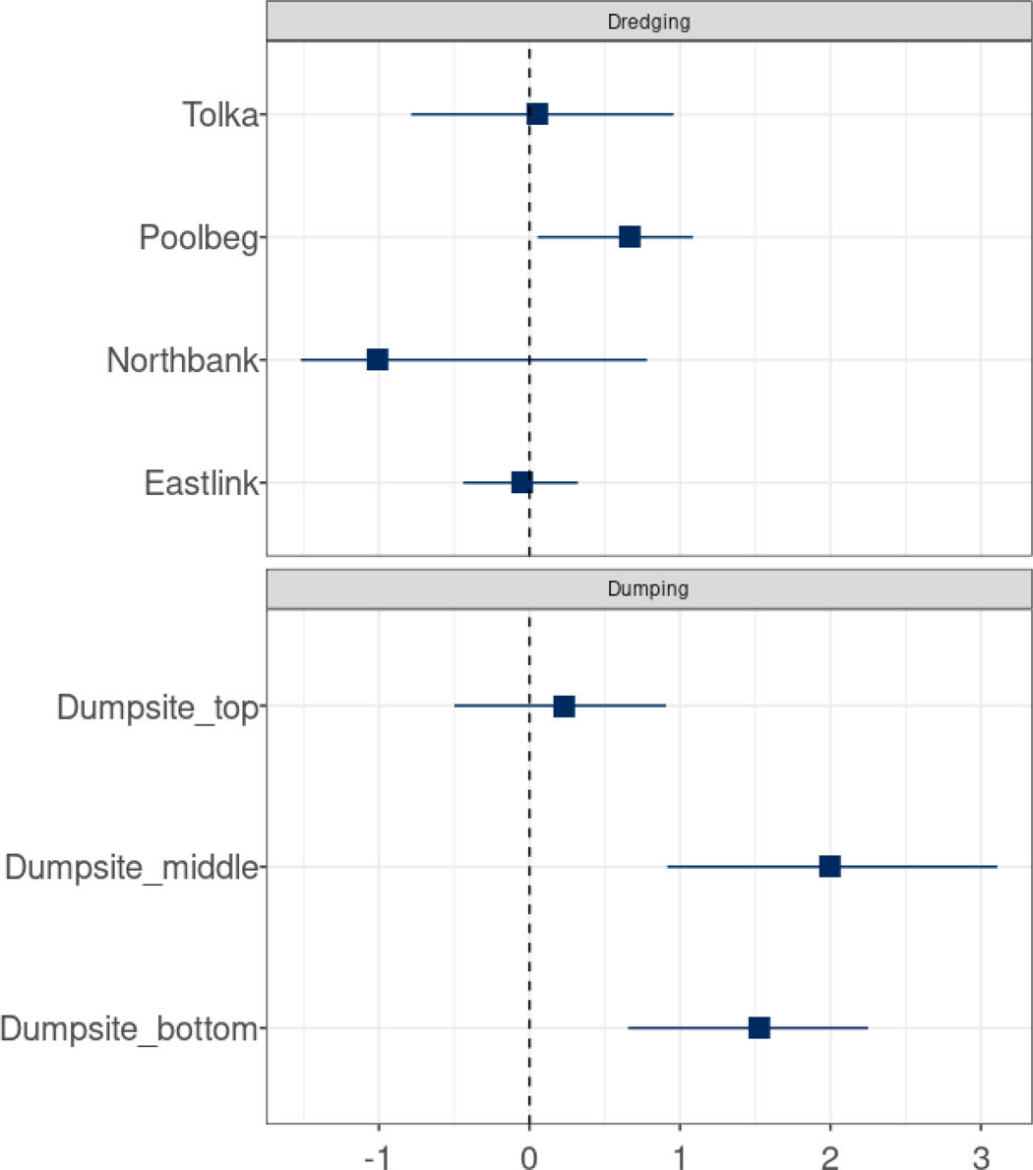
Dumping and dredging effects (NTU/day) at different locations with the 95% credible interval.

To determine the degree to which dumping and dredging operations affect turbidity, we evaluate the posterior distribution of the fixed effects *β*. Figure 6 shows the expected value of the dumping and dredging effects respectively with their 95% credible intervals for different locations. Most effects are observed to be close to zero. According to the figure, dumping at the middle depth of the dumpsite has the most significant positive impact, followed by the effect of dumping at the bottom of the dumpsite.

**Figure 7.**
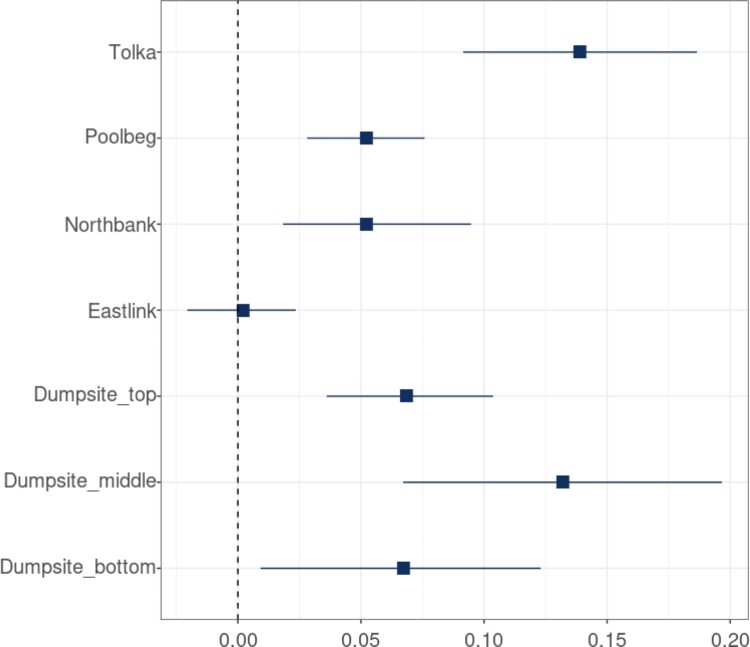
Effect of wind speed (NTU/Knot) at different locations and depths with the 95% credible interval.

Figure 7 shows the wind speed effects for the 7 buoys. These are measured in NTU per knot and these wind effects can be more clearly identified than the effects of dredging and dumping. The values are reasonably consistent but with greater uncertainty at the lower positions in the dumping buoy, and a far smaller effect at Eastlink, again likely due to its position in the bay. By contrast, the Tolka buoy seems most influenced by wind and is the site that is most far out to sea. The Tolka buoy is situated within the confines of the estuary walls, adjacent to North Bull wall. This area of the estuary is relatively shallow and at low tide is exposed to the wind.

### 5.3. Influence of the autoregressive component

**Figure 8.**
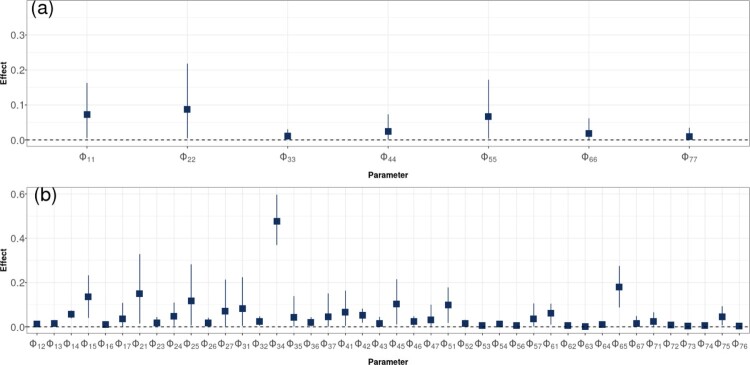
Coefficients of the Φ matrix with their 95% credible interval. Diagonal values are shown in the top panel (a) and off-diagonal values are shown in (b). The two subscripts indicate the parent and child relationship respectively, so that $\Phi_{12}$ for example is the degree to which buoy 2 influences the time series of buoy 1. The numbers of the buoys follow the labelling defined in Figure 1.

As a final part of the analysis, we examine the autoregressive coefficients from the VARICH model. Figure 8 shows the posterior coefficients of Φ where we have separated out the diagonal values which indicate the influence of the time series on itself from the off-diagonal elements which show the influence of one site on another. The numbering of the sites is as shown in Figure 1.

Of the diagonal elements, the dumpsite (middle) seems to have the most dependence after accounting for the integration component. The other sites have values close to zero after accounting for uncertainty. Of the off-diagonal elements, some of these are well away from zero and provide for interesting, if not entirely straightforward, interpretation. $\Phi_{34}$ is the largest, corresponding to the relationship between dumpsite (bottom) and buoy 4 (Tolka), which should perhaps be read in conjunction with their joint time series behaviour as shown in Figure 4. Many of the other off-diagonal elements show similar clear non-zero effect sizes though they are considerably smaller than $\Phi_{34}$. These values provide evidence of cross site learning in the time series model.

## 6. Conclusions

We have introduced a set of models for understanding the behaviour of turbidity in Dublin bay. Both the VARCH and the VARICH models introduced in Section 4 allow for measuring the effects of multivariate time series on each other, whilst taking account of the known volatility changes in the time series. However, the VARICH model had slightly better performance. The combination of Bayesian modelling, VAR and ARCH structures makes the VARICH model a useful tool for flexible modelling of a wide range of real world random processes in which spatial and temporal aspects are playing major roles. Furthermore, the Bayesian approach allows for uncertainty quantification of both the fixed effects and the posterior predictions of the time series, whilst simultaneously imputing the missing values within the series.

Our main finding has been that the dumping and dredging operations have minimal effect on the turbidity levels, which seem to be more affected by wind speed and previous values of the series. We thus suggest that, at an aggregate daily level, there is minimal effect of dredging on the turbidity levels in Dublin bay. The models we produced seem to fit the data well and the results make physical sense according to the location of the buoys in the bay. A longer time series and a more complete record would add further weight to our conclusions.

Our model fitting technique of using HMC appeared to converge efficiently and quickly on a standard laptop, taking around 10 min to reach R-hat values below the common standard of 1.1 whilst requiring only 4 chains of 1000 posterior draws (with 200 removed during the warm-up phase). However, for larger data sets it may be that users need to increase the number of draws. For very large data sets the HMC technique may prove infeasible and so other methods such as MultiBUGS [14] might be more appropriate. Other computational difficulties may be occur should the model structure be made more complex. Interesting extensions of our approach might involve looking at time-varying behaviour of the coefficients, or multiple lags or long memory of the multivariate time series itself.

## Code availability

The codes used in this study are available on Github (https://github.com/Aminsn/VARICH2023, last access: 27 September 2023).

## Appendix 1

**Table B1.** Summary statistics of estimated parameters, including their Mean, Median, Standard Deviation, 5th percentile, 95th percentile, and the R-hat statistic.

Variable	Mean	Median	SD	q5	q95	Rhat
beta1[1]	0.07	0.07	0.02	0.04	0.1	1
beta1[2]	0.13	0.13	0.03	0.08	0.19	1
beta1[3]	0.07	0.07	0.03	0.02	0.11	1
beta1[4]	0.14	0.14	0.02	0.1	0.18	1
beta1[5]	0.05	0.05	0.02	0.02	0.08	1
beta1[6]	0.05	0.05	0.01	0.03	0.07	1
beta1[7]	0	0	0.01	−−0.02	0.02	1
beta2[1]	0.26	0.27	0.33	−−0.29	0.8	1
beta2[2]	1.99	1.98	0.56	1.08	2.94	1
beta2[3]	1.54	1.57	0.41	0.84	2.16	1
beta2[4]	0.04	0	0.48	−−0.69	0.86	1
beta2[5]	−−1.08	−−1.08	0.24	−−1.46	−−0.69	1
beta2[6]	0.68	0.71	0.26	0.22	1.04	1
beta2[7]	−−0.04	−−0.04	0.19	−−0.37	0.27	1
alpha[1]	2.78	2.85	0.54	1.87	3.52	1.01
alpha[2]	4.3	4.32	0.28	3.83	4.73	1
alpha[3]	3.8	3.79	0.16	3.55	4.08	1
alpha[4]	1.22	1.25	0.36	0.59	1.75	1.01
alpha[5]	−−1.46	−−1.43	0.33	−−2.05	−−0.96	1
alpha[6]	0.42	0.43	0.13	0.21	0.64	1.01
alpha[7]	−−2.05	−−2.01	0.52	−−2.97	−−1.26	1.01
gamma0[1]	0.69	0.68	0.1	0.53	0.87	1
gamma0[2]	2.09	2.08	0.33	1.56	2.67	1
gamma0[3]	1.14	1.02	0.52	0.53	2.17	1
gamma0[4]	1.65	1.64	0.21	1.33	2.01	1
gamma0[5]	0.09	0.06	0.09	0	0.27	1
gamma0[6]	0.2	0.2	0.03	0.15	0.26	1.01
gamma0[7]	0.07	0.05	0.06	0	0.19	1
gamma1[1]	0.07	0.07	0.02	0.05	0.1	1.01
gamma1[2]	0.38	0.38	0.05	0.3	0.47	1
gamma1[3]	0.62	0.63	0.09	0.45	0.76	1
gamma1[4]	0.44	0.44	0.07	0.33	0.57	1.01
gamma1[5]	0.13	0.13	0.03	0.1	0.18	1
gamma1[6]	0.55	0.55	0.05	0.47	0.64	1
gamma1[7]	0.05	0.05	0.01	0.03	0.07	1
phi[1,1]	0.08	0.07	0.04	0.01	0.15	1
phi[2,1]	0.15	0.14	0.08	0.03	0.3	1
phi[3,1]	0.08	0.07	0.06	0.01	0.19	1
phi[4,1]	0.06	0.06	0.04	0.01	0.15	1.01
phi[5,1]	0.1	0.1	0.04	0.03	0.16	1.03
phi[6,1]	0.06	0.06	0.02	0.02	0.1	1.03
phi[7,1]	0.02	0.02	0.02	0	0.06	1
phi[1,2]	0.01	0.01	0.01	0	0.02	1.01
phi[2,2]	0.08	0.08	0.06	0.01	0.19	1
phi[3,2]	0.02	0.02	0.01	0.01	0.04	1.01
phi[4,2]	0.05	0.05	0.02	0.02	0.08	1.02
phi[5,2]	0.01	0.01	0.01	0	0.03	1.01
phi[6,2]	0	0	0	0	0.01	1.01
phi[7,2]	0.01	0.01	0	0	0.02	1.01
phi[1,3]	0.02	0.01	0.01	0	0.03	1
phi[2,3]	0.02	0.02	0.01	0	0.04	1
phi[3,3]	0.01	0.01	0.01	0	0.03	1
phi[4,3]	0.01	0.01	0.01	0	0.04	1
phi[5,3]	0.01	0	0	0	0.01	1
phi[6,3]	0	0	0	0	0.01	1
phi[7,3]	0	0	0	0	0.01	1
phi[1,4]	0.06	0.06	0.01	0.04	0.07	1
phi[2,4]	0.05	0.05	0.03	0.01	0.1	1
phi[3,4]	0.48	0.47	0.05	0.39	0.57	1
phi[4,4]	0.02	0.02	0.02	0	0.06	1
phi[5,4]	0.01	0.01	0.01	0	0.02	1
phi[6,4]	0.01	0.01	0	0	0.02	1
phi[7,4]	0.01	0.01	0	0	0.01	1
phi[1,5]	0.13	0.14	0.05	0.05	0.22	1
phi[2,5]	0.12	0.11	0.08	0.01	0.26	1
phi[3,5]	0.05	0.03	0.04	0	0.12	1
phi[4,5]	0.11	0.1	0.05	0.02	0.2	1.01
phi[5,5]	0.07	0.06	0.04	0.01	0.15	1
phi[6,5]	0.18	0.18	0.05	0.1	0.26	1
phi[7,5]	0.05	0.04	0.02	0.01	0.08	1
phi[1,6]	0.01	0.01	0.01	0	0.02	1
phi[2,6]	0.02	0.02	0.01	0	0.04	1
phi[3,6]	0.02	0.02	0.01	0	0.04	1
phi[4,6]	0.02	0.02	0.01	0.01	0.04	1
phi[5,6]	0.01	0	0	0	0.02	1
phi[6,6]	0.02	0.01	0.02	0	0.05	1
phi[7,6]	0	0	0	0	0.01	1
phi[1,7]	0.04	0.03	0.03	0	0.09	1
phi[2,7]	0.07	0.06	0.06	0	0.18	1
phi[3,7]	0.05	0.03	0.04	0	0.12	1
phi[4,7]	0.03	0.02	0.03	0	0.08	1
phi[5,7]	0.04	0.03	0.03	0	0.09	1
phi[6,7]	0.02	0.01	0.01	0	0.04	1
phi[7,7]	0.01	0.01	0.01	0	0.03	1

## Appendix 2

To fit a frequentist VAR (Vector Autoregression) model to our data, we utilised the ‘vars‘ package in R. This package does not accommodate missing values in the data, and to the best of our knowledge, there is no package that can naturally handle missing data without resorting to imputation techniques. Therefore, we first addressed this issue by imputing the missing values using the forward-fill method, whereby we carried the last observed values forward to replace any missingness. After fitting the model, we calculated the root mean squared error (RMSE) for one-step-ahead predictions. We then compared these results with those obtained from the VARICH model, as detailed in Table A3. Please note that calculating the WAIC and LOOIC criteria to compare the two models, as was done in Figure 3, is not possible because these calculations require evaluating the log-likelihood over the predictive posterior distribution, which is unavailable in the frequentist paradigm. As presented in Table A3, the VARICH model's RMSE values are lower for all sites except Poolbeg, where both models performed the worst, possibly due to the highest volatility among the sites.

**Table C1.** Comparison of RMSE values: VARICH (Bayesian) vs VAR (Frequentist).

Variable	VARICH	VAR
Buoy 1 Top	**1.89**	2.08
Buoy 1 Middle	**5.14**	8.31
Buoy 1 Bottom	**5.69**	6.65
Tolka	**4.26**	4.30
Northbank	**2.40**	2.45
Poolbeg	8.94	**8.36**
Eastlink	**1.19**	1.22
